# Massive adrenal vein aneurysm mimicking an adrenal tumor in a patient with hemophilia A: a case report and review of the literature

**DOI:** 10.1186/s13256-016-1108-z

**Published:** 2016-12-01

**Authors:** Richard Sleightholm, Steven Wahlmeier, Jeffrey S. Carson, Andjela Drincic, Audrey Lazenby, Jason M. Foster

**Affiliations:** 1 Division of Surgical Oncology, Department of Surgery, University of Nebraska Medical Center, 986345 Nebraska Medical Center, Omaha, NE 68198-6345 USA; 2 Division of Endocrinology, Department of Medicine, University of Nebraska Medical Center, Omaha, NE USA; 3 Department of Pathology and Microbiology, University of Nebraska Medical Center, Omaha, NE USA

**Keywords:** Adrenal artery aneurysm, Hemophilia, Case report

## Abstract

**Background:**

Visceral venous aneurysms are exceedingly rare, and until now, there have been no reports of this phenomenon in the adrenal vasculature. This report details the first adrenal venous aneurysm reported in the literature. The aneurysm presented as an 18-cm mass that was initially suspected to be a hematoma or tumor on the basis of the complex medical history of the patient, which included hemophilia A and testicular cancer. After surgical excision, pathologic examination confirmed this mass to be a 15.9-cm adrenal vein aneurysm, the largest aneurysm of any type or location recorded in the medical literature.

**Case presentation:**

A 58-year-old caucasian male with hemophilia A presented to the emergency room of another institution with abdominal pain, blood in the stool, and a history of diverticulosis and symptomatic hemorrhoids. A large, left-sided adrenal mass was detected by computed tomography, and because of the patient’s hemophilia A and imaging consistent with a hemorrhagic mass, a hematoma was initially suspected. The patient was transferred to our institution, monitored for further bleeding with a stable hospital course, and discharged from the hospital under close monitoring. After 7–8 weeks with no change in the size of the mass, concerns grew regarding increasing symptoms of both satiety and mass effects from the large anomaly, as well as about the patient’s complicated medical history, which also included cancer. Surgical excision was recommended because of the concerns about increasing symptoms and the possibility of a malignancy. Correction and maintenance of factor VIII levels were incorporated pre-, intra-, and postoperatively, and *en bloc* surgical resection was performed to minimize bleeding and provide oncologic extirpation of the mass. A bowling ball-sized mass was removed, and careful pathologic examination revealed the mass to be a venous adrenal aneurysm. After a brief hospital stay, the patient made a full recovery. Extensive review of the literature revealed 11 reports of adrenal artery aneurysms but no reported case of an adrenal aneurysm arising from the venous system.

**Conclusions:**

Several case reports suggest a correlation between hemophilia and aneurysms. In patients with inherited clotting disorders such as hemophilia A, aneurysms may present in atypical fashions and should be carefully ruled out.

## Background

Adrenal artery aneurysms are rare, with only 11 cases previously reported in the literature (Table 1). These patients often present late because of symptoms due to life-threatening rupture and hemodynamic instability. There has been no reported incidence of a venous aneurysm arising from the adrenal vasculature or in conjunction with a coagulation disorder. We report a case of a 58-year-old white man who underwent surgical removal of a densely adherent abdominal mass compressing visceral organs that was initially thought to be a hematoma as a result of his hemophilia A or potential malignant process due to his history of testicular cancer. Pathologic examination revealed a 15.9-cm, atypical adrenal venous aneurysm. This represents, to the best of our knowledge, the first reported venous adrenal aneurysm and the first venous aneurysm reported in a patient with hemophilia A. Interestingly, this also appears to be the largest aneurysm reported in the literature. Furthermore, this report describes the details of an aneurysm of the adrenal venous vasculature in a patient with hemophilia A and how the combination of the low-pressure system of the adrenal vein and the patient’s hemophilic physiology may have contributed to the large size of the aneurysm at presentation.

**Table 1:** Literature review of all adrenal aneurysms

First author, year [reference]	Patient age, years	Sex	Side	Comorbidities	Complications	Mortality
Wiseman, 2013 [5]	80	M	R	None	None	No
Bolla, 2012 [6]	28	F	R	Pregnancy, labor	None	No
Glocker, 2011 [7]	33	F	L	Neurofibromatosis, pheochromocytoma	None	No
Manners, 2010 [8]	70	M	L	Hypertension	None	No
González Valverde, 2007 [4]	42	M	L	Hypertension	None	No
Christie, 2004 [13]	32	F	L	Pregnancy, labor	None	No
Nakano, 2003 [9]	68	M	L	Hypertension, renal insufficiency	Renal failure requiring dialysis	No
Park, 2003 [14]	32	M	R	Bilateral pheochromocytoma	None	No
Bromley, 2001 [10]	27	F	R	Unknown	none	No
Thanos, 2000 [11]	75	M	L	None	None	No
Birchall, 1995 [12]	62	M	R	None	none	No

Eleven reports that described adrenal aneurysms, all of which were arterial, were found in the literature; no venous aneurysms were noted

*M* Male, *F* Female, *L* Left, *R* Right

## Case presentation

A 58-year-old white man presented to an outside hospital for evaluation of bright red blood hemorrhaging from the rectum. In the course of the workup, an adrenal mass was found incidentally by abdominal computed tomography (CT). The patient’s past medical history included hemophilia A, type 2 diabetes mellitus, and testicular cancer. His surgical history was significant for cholecystectomy and tonsillectomy. The patient’s initial physical examination was benign with the exception of a large, firm, palpable, nonpulsating, nontender mass in the upper left quadrant of the abdomen. His baseline factor VIII activity level was 12%. While the patient was being evaluated in the hospital, he received 3500 U of recombinant factor VIII (Advate; Shire/Baxter Healthcare, Westlake Village, CA, USA) twice daily with a goal factor VIII activity level between 80% and 100%.

**Fig. 1:**
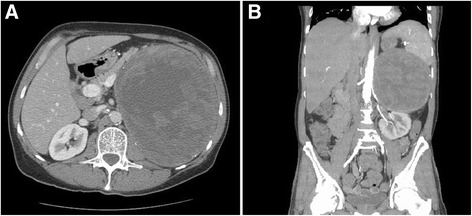
**a** Axial cross-section depicts the displacement of the stomach anteromedially. **b** Coronal cross-section shows the aneurysm displacing both the spleen superiorly and the kidney inferiorly. Collectively, these two images illustrate that the mass has grown to occupy a vast majority of the entire left upper quadrant

The initial CT scan of the patient revealed a large, heterogeneous, hemorrhagic mass in the suprarenal fossa causing mass effect on the stomach, pancreatic tail, and left kidney (Fig. 1). The mass was estimated to be 18 × 17 × 16 cm in size. The differential diagnosis included hemorrhagic adrenal malignancy or benign tumor vs. pure hemorrhage or hematoma of the adrenal gland. There were no signs or symptoms of hormone overproduction. The patient was normotensive, not tachycardic, was normokalemic, and had no cushingoid symptoms or appreciable gynecomastia. The patient underwent a comprehensive endocrinologic workup with biochemical evaluation for a functional adrenal tumor, including plasma metanephrine (<0.20 nmol/L), normetanephrine (0.22 nmol/L), 17-hydroxyprogesterone (74.6 ng/dl), estradiol (30 pg/ml), dehydroepiandrosterone sulfate (43 μg/dl), a dexamethasone suppression test (14.4 μg/dl at 0 minutes, 23.3 μg/dl at 30 minutes, and 26.1 μg/dl at 60 minutes), adrenocorticotropic hormone (27 pg/ml), aldosterone (3 ng/dl), renin activity (8.5 ng/ml/h), and androstenedione level (0.215 ng/ml). The biochemical evaluation showed no evidence of an occult functional adrenal lesion or condition.

After a more detailed history focused on trauma, the patient recalled that he had sustained a slight fall in the previous weeks that provided a mechanism for the mass, most consistent with a hematoma. The patient was discharged from the hospital, and the adrenal mass was monitored on an outpatient basis. A repeat CT scan at 4 weeks following diagnosis did not demonstrate any change in the size of the mass, and the patient had no appreciable changes in signs or symptoms. At around the seventh week, the patient reported increasing symptoms of early satiety and left upper quadrant pain, but repeat imaging did not reveal any enlargement of the mass. The patient was taken to the operating room for *en bloc* resection, and, given the patient’s history of hemophilia A, he required extra monitoring and management perioperatively. Specifically, he received 2–4 U/kg/h of recombinant factor VIII, and his factor VIII activity was monitored to maintain an activity level ≥80%.

**Fig. 2:**
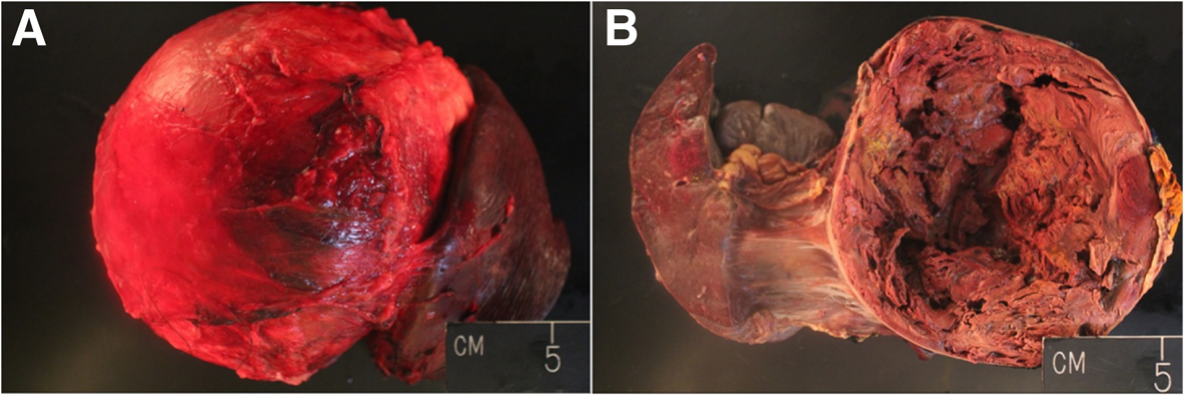
**a** Gross pathological specimen displays significant encasement of the mass in fibrotic material. **b** Bisected view of the vessel with atypical architecture adrenal venous aneurysm

Through a midline approach, the mass was visualized and found to displace the left kidney inferiorly, the spleen superiorly, the pancreas laterally, and the stomach anteromedially. The mass was adherent to the surrounding structures, including the adrenal gland, the tail of the pancreas, and the capsule of the spleen, without any definable plains of dissection, necessitating removal of 40% of the pancreas, spleen, adrenal gland, and a portion of the diaphragm. The mass was removed and sent for evaluation in the pathology department (Fig. 2a). Given the abutment of midline vascular structures, including portal vein, aorta, and vena cava, a postresection intraoperative ultrasound was obtained to confirm the patency of these structures, and no occult thrombosis was detected. Esophagogastroduodenoscopy was performed given the gastric abutment and extensive greater curvature/short gastric dissection and revealed normal gastric mucosa.

The postoperative mass was measured to be 20 cm. The final pathological analysis determined the specimen to be an adrenal vein aneurysm consisting mostly of thrombotic material with a conservative measurement of 15.9 cm (Fig. 2b). The wall of the vessel itself was comprised mainly of thick smooth muscle with some elastic fibers and no elastic lamina. The adrenal gland was adherent to the mass and was benign. All of the *en bloc* surgical specimens were benign.

Postoperatively, the patient’s factor VIII activity level was maintained with a goal of 60–80% in the first 3 days and a goal of 50% for the first 2 weeks. His postoperative hospital course was complicated by a low-output pancreatic leak, for which he was discharged with percutaneous drains in the pancreatectomy bed and on total parenteral nutrition with clear liquids for comfort. The patient made a full recovery from his pancreatic complications approximately 4 weeks postoperatively, and he was reevaluated with CT at 6 months postoperatively with postsurgical changes of absence of spleen, 40% of pancreas, adrenal gland, and absence of the adrenal mass. At 23 months of postoperative follow-up, the patient was doing well without any new issues.

## Discussion

Visceral artery aneurysms (VAAs) are uncommon, and visceral venous aneurysms (VVAs) are rare and therefore much less described. The overall incidence of VAAs found on autopsy is reported at 0.01–0.2%, with the most common locations being the splenic, hepatic, and celiac arteries [1]. One hundred ninety-nine VVA cases have been published, with the portal venous system accounting for over 95% of these reported occurrences [2, 3]. Our literature search revealed a limited number of aneurysms arising in the adrenal vasculature, with only 11 cases previously reported and all occurring in the adrenal artery [4–14]. To the best of our knowledge, this is the first report of an aneurysmal formation arising from the venous vasculature of the adrenal gland and the first case of an adrenal vasculature anomaly in a patient with hemophilia A. Moreover, this case represents the largest known aneurysm from all vascular sites reported in the literature [2, 3].

Recognized medical risk factors predisposing to aneurysm development include tobacco use, atherosclerosis, congenital malformations, connective tissue disorders, autoimmune mechanisms, local inflammatory conditions, infections, primary arterial injury/trauma, vasculitis, medial fibrodysplasia, hypertension, and pregnancy [4]. The most concerning and life-threatening sequelae are rupture and hemorrhage. Most VAAs are asymptomatic and found incidentally or only after rupture occurs [15]. In the instance of a VAA or VVA rupture, diagnosis may be delayed because of inability to locate the origin of the bleed and technical difficulty in establishing rapid hemostasis, often with life-threatening hemorrhage. Rupture in patients with inherited coagulation disorders presents an additional layer of challenge, not only in the setting of hemorrhage but also in the management of surgical bleeding risks. Currently, the presence of therapies such as concentrated or recombinant factor VIII for patients with hemophilia A has decreased these risks [16, 17]. The fact remains, however, that these patients present unique surgical challenges: evaluating and correcting pre-, intra-, and postoperative hemostasis; minimizing blood loss intraoperatively; and decreasing the risk of bleeding from the surgical sites postoperatively [18].

We report a very unique case of a patient with hemophilia A who presented with a large abdominal mass of unknown origin. Important considerations for this patient were, first, establishing a diagnosis and, second, appropriate management of the mass in the setting of increased bleeding risk. At the time of the operation, there was no hemoperitoneum, and a solid mass raising concern for malignancy was appreciable. The specimen was densely adherent to the surrounding tissue, which required meticulous adhesiolysis and *en bloc* resection of the left adrenal gland, distal pancreas, spleen, and a small portion of the diaphragm. The pathology report described a thickly muscled vessel devoid of elastic lamina and containing thrombotic material consistent with an adrenal vein aneurysm. Interestingly, this patient developed atypical symptoms (early satiety and left upper quadrant fullness/pain) that arose from mass effect due to the size of the aneurysm and not from the hemodynamic instability of rupture. To date, authors of 11 other case reports have described aneurysms of the adrenal vasculature, with none of these cases arising from the adrenal vein (Table 1). All but one individual presented with a ruptured aneurysm resulting in hemodynamic instability. The other patient was admitted for symptoms arising from a pheochromocytoma, and an incidental adrenal artery aneurysm was detected at the time of laparoscopic adrenalectomy.

Several reports have described patients with hemophilia with various aneurysmal formations [18–23]. Given the atypical presentation of our patient, his bleeding disorder may have precipitated and/or exacerbated the aneurysm formation. Furthermore, authors of many case reports have depicted abnormal aneurysmal formations in patients with hemophilia A, such as a radial artery aneurysm [20]. Because visceral aneurysms are uncommon and hemophilia is rare, epidemiologic studies to examine true statistically significant correlations would be particularly challenging and not practical. Thus, accumulation of case reports such as these are crucial to providing preliminary insight into uncommon disease events such as aneurysms in the presence of rare genetic conditions such as hemophilia.

We report a case of a patient with hemophilia and an aneurysmal formation who presented with atypical symptoms masquerading as an adrenal mass with associated hematoma on CT and magnetic resonance imaging, which was likely due to (1) the location of the mass in the adrenal glands, (2) the mass being a venous instead of an arterial malformation, and (3) the large size of the mass. Because of these properties in conjunction with the patient’s medical history of hemophilia and cancer history, an oncologic process (primary adrenal vs. metastatic) with traumatic bleeding was considered the most likely diagnosis, especially with persistence of the mass after 6–7 weeks of observation. In retrospect, now that we know that this was a venous aneurysm, we speculate that duplex vascular flow studies may have provided useful information. We are more inclined to screen for such vascular abnormalities in the future in patients with coagulation disorders such as hemophilia who present with masses of unknown origin. We recommend that other healthcare providers be aware of a potential correlation and consider pursuing noninvasive testing such as duplex ultrasound.

The entire mass measured 20 cm, whereas the venous aneurysm component was reported by the pathologist to be 15.9 cm and comparable in size to the average bowling or soccer ball. This appears to be the largest known and surgically managed aneurysm of any type in the literature. A report published in the 1960s described one of the largest reported aneurysms, 11 cm, arising from the internal iliac artery [24]. Another report, published in 2008, described an 11.8-cm external iliac vein aneurysm originating from an old stab wound the patient had sustained 20 years prior [25]. Finally, the largest aneurysm on record measured 11.7 cm arising from the ascending aorta and was successfully resected and repaired [26].

## Conclusions

Our patient presented with a challenging and unique case, clinically and surgically, that required multidisciplinary evaluation by the surgery, endocrinology, hematology, and pathology departments. Complex surgical produres can be performed safely on patients with coagulation disorders such as hemophilia A can be done safely with available medical resources of recombinant factors and guidelines for monitoring factor replacement to reduce the risk of perioperative bleeding. Visceral aneurysms are rare, and formation of aneurysms in the lower-pressure venous system of the adrenal vasculature at such a large size as that in our patient is unprecedented. To the best of our knowledge, we report the first case in the literature of an adrenal venous aneurysm as well as the largest known aneurysmal formation. Visceral aneurysms can occur in hemophiliacs, and aneurysms in this patient population should remain in the differential diagnosis in an effort to identify them prior to rupture, given the challenges of managing acute bleeding in this population. Conversely, embolism of the thrombotic material is an additional sequela of visceral aneurysm that can result in organ ishemia, organ loss, and life threatening illness. Although these visceral aneurysms are rare, rates of rupture remain high. It is imperative that providers be aware of this diagnosis and implement efforts to rule out an aneurysm by diagnostic means such as radiographic imaging and duplex ultrasound.

## Abbreviations

CT: Computed tomography
GI: Gastrointestinal
VAA: Visceral artery aneurysm
VVA: Visceral vein aneurysm

## References

[1] Chadha M, Ahuja C. Visceral artery aneurysms: diagnosis and percutaneous management. Semin Interv Radiol. 2009;26(3):196–206. 10.1055/s-0029-1225670 PMC3036493 21326564

[2] Ibrahim WH, Bassurrah HM. Endovascular management of splenic arteriovenous fistula with giant venous aneurysmal dilatation. Ann Vasc Dis. 2012;5(4):439–44. 10.3400/avd.cr.12.00009 PMC3641543 23641267

[3] Sfyroeras GS, Antoniou GA, Drakou AA, Karathanos C, Giannoukas AD. Visceral venous aneurysms: clinical presentation, natural history and their management: a systematic review. Eur J Vasc Endovasc Surg. 2009;38(4):498–505. 10.1016/j.ejvs.2009.05.016 19560947

[4] González Valverde FM, Balsalobre M, Torregrosa N, Molto M, Gómez Ramos MJ, Vázquez Rojas JL. Spontaneous retroperitoneal hemorrhage from adrenal artery aneurysm. Cardiovasc Intervent Radiol. 2007;30(2):307–9. 10.1007/s00270-006-0029-4 16967216

[5] Wiseman D, Harris K, Ehmann J. Spontaneous rupture of a rare adrenal artery aneurysm mimicking a ruptured abdominal aortic aneurysm. Vasc Endovasc Surg. 2013;47(2):159–62. 10.1177/1538574412470740 23275484

[6] Bolla D, Schyrba V, Drack G, Dietler S, Hornung R. Spontaneous rupture of an adrenal artery in pregnancy: a case report. Case Rep Obstet Gynecol. 2012;2012:859068. 10.1155/2012/859068 PMC3546435 23346435

[7] Glocker R, Ruan DT, Gillespie D, Wittlin S, Dombrowski D, Moalem J. Adrenal artery aneurysm encountered during laparoscopic adrenalectomy for pheochromocytoma. J Vasc Surg. 2011;54(1):216–8. 10.1016/j.jvs.2010.11.128 21334168

[8] Manners J, Singh R, Page A, Adamson A, McLean D. Radiological treatment of a spontaneously ruptured inferior adrenal artery aneurysm. Nat Rev Urol. 2010;7(12):694–8. 10.1038/nrurol.2010.184 21045799

[9] Nakano M, Takada T, Takahashi Y, Ishihara S, Kondo H, Deguchi T. Spontaneous ruptured adrenal artery aneurysm. J Urol. 2003;169(2):614. 10.1097/01.ju.0000042460.57893.e6 12544322

[10] Bromley PJ, Balich SM, Giddens C, Halpern NB, Barton RE, Keller FS. SCVIR annual meeting film panel session: diagnosis and discussion of case 3: right middle adrenal artery aneurysm. J Vasc Interv Radiol. 2001;12(4):540–3. 10.1016/s1051-0443(07)61899-5 11287547

[11] Thanos L, Papaioannou G, Grammenou-Pomoni M, Malagari K, Brountzos EN, Kelekis D. Ruptured adrenal artery aneurysm: a case report. Eur Radiol. 2000;10(1):105–7. 10.1007/s003300050013 10663724

[12] Birchall D, Carney AS, Morse MH. Case report: ruptured adrenal artery aneurysm. Clin Radiol. 1995;50(10):732–3. 10.1016/s0009-9260(05)83325-2 7586972

[13] Christie J, Batool I, Moss J, Macara L. Adrenal artery rupture in pregnancy: a case report. BJOG. 2004;111(2):185–7. 10.1046/j.1471-0528.2003.00047.x 14723762

[14] Park J, Ang KP, Lee SJ, Kim CH, Park TS, Baek HS. A case of a ruptured pheochromocytoma with an intratumoral aneurysm managed by coil embolization. Endocr J. 2003;50(6):653–6. 10.1507/endocrj.50.653 14709833

[15] Pitton MB, Dappa E, Jungmann F, Kloeckner R, Schotten S, Wirth GM, *et al*. Visceral artery aneurysms: incidence, management, and outcome analysis in a tertiary care center over one decade. Eur Radiol. 2015;25(7):2004–14. 10.1007/s00330-015-3599-1 PMC4457909 25693662

[16] Brown B, Steed DL, Webster MW, Makaroun MS, Spero JA, Bontempo FA, *et al*. General surgery in adult hemophiliacs. Surgery. 1986;99(2):154–9. 3080817

[17] Ferraris VA, Boral LI, Cohen AJ, Smyth SS, White GC. Consensus review of the treatment of cardiovascular disease in people with hemophilia A and B. Cardiol Rev. 2015;23(2):53–68. 10.1097/CRD.0000000000000045 PMC4323575 25436468

[18] Kobayashi M, Matsushita M, Nishikimi N, Sakurai T, Takamatsu J, Nimura Y. Treatment for abdominal aortic aneurysm in a patient with hemophilia A: a case report and review of the literature. J Vasc Surg. 1997;25(5):945–8. 10.1016/s0741-5214(97)70228-3 9152326

[19] Diplaris KT, Karfis EA, Ampatzidou F, Ananiadou OG, Vakalopoulou S, Madesis A, *et al*. Acute type-A dissection in a patient with severe hemophilia A. J Cardiothorac Vasc Anesth. 2012;26(4):660–3. 10.1053/j.jvca.2011.07.017 21937244

[20] Filis K, Arhontovassilis F, Theodorou D, Theodossiades G, Manouras A. True radial artery aneurysm in a mild haemophilia A patient. Haemophilia. 2007;13(4):440–2. 10.1111/j.1365-2516.2007.01465.x 17610564

[21] Wiszniewski A, Szopiński P. Surgical treatment of arteriovenous fistula and brachial artery aneurysm in a patient with mild haemophilia A. Haemophilia. 2011;17(3):548–50. 10.1111/j.1365-2516.2010.02425.x 21299738

[22] Sadat U, Naik J, Hayes PD. Surgical complications in a hemophilia patient with factor VIII inhibitor and their endovascular management. Vasc Endovasc Surg. 2008;42(2):168–72. 10.1177/1538574407309319 18421033

[23] Mann HA, Goddard NJ, Lee CA, Brown SA. Periarticular aneurysm following total knee replacement in hemophilic arthropathy: a case report. J Bone Joint Surg Am. 2003;85-A(12):2437–40. 10.2106/00004623-200312000-00025 14668517

[24] Frank I, Thompson H, Rob C, Schwartz S. Aneurysm of the internal iliac artery. Arch Surg. 1961;83(6):956–8. 10.1001/archsurg.1961.01300180156030 13894626

[25] Kuhlencordt P, Linsenmeyer U, Rademacher A, Sadeghi-Azandaryani M, Steckmeier B, Hoffmann U. Large external iliac vein aneurysm in a patient with a post-traumatic femoral arteriovenous fistula. J Vasc Surg. 2008;47(1):205–8. 10.1016/j.jvs.2007.07.060 18178475

[26] Guinness world record. http://www.guinnessworldrecords.com/world-records/largest-aneurysm-removed.

